# Sound touch elastography for assessing cirrhosis preoperatively in infants with biliary atresia: Comparison with serum fibrosis biomarkers

**DOI:** 10.3389/fped.2022.989293

**Published:** 2022-09-29

**Authors:** Xingxing Duan, Liu Yang, Rong Wen, Hong Cao, Huan Wen, Wengang Liu, Hongxia Yuan

**Affiliations:** ^1^Department of Ultrasonography, Changsha Hospital for Maternal and Child Health Care, Changsha, China; ^2^Department of Ultrasound, Hunan Children’s Hospital, Changsha, China; ^3^Department of Pathology, Hunan Children’s Hospital, Changsha, China; ^4^Department of Ultrasound, The Third Xiangya Hospital of Central South University, Changsha, China

**Keywords:** biliary atresia (BA), ultrasound, elastography, hepatic fibrosis, cirrhosis

## Abstract

**Introduction:**

The accurate assessment of the stages of hepatic fibrosis in children with biliary atresia (BA) before performing Kasai portoenterostomy (KPE) is of utmost importance. Some studies demonstrated that ultrasound elastography can be used to assess the stages of hepatic fibrosis by detecting liver stiffness. Therefore, the aim of this work was to explore the usefulness of sound touch elastography (STE) for preoperatively assessing liver cirrhosis in infants with BA.

**Methods:**

A total of 189 children from the Hunan Children’s Hospital with highly suspected BA were selected for this study, and their preoperative liver STE values and related clinical data were collected. The pathological results of the liver stages were considered as the gold standard. Spearman correlation was used to analyze the correlation between each parameter and the stage of hepatic fibrosis, and the receiver operator characteristic (ROC) curve was used to analyze the diagnostic performance for cirrhosis of each parameter.

**Results:**

Among the selected 189 patients with suspected BA, 159 were included in this study and were composed of 3 at hepatic fibrosis stage F1, 45 at stage F2, 41 at stage F3, and 70 at stage F4, while no patients at stage F0 were present. Spearman correlation analysis showed that the liver STE value had the highest correlation with the stage of hepatic fibrosis, with a correlation coefficient of 0.813 (*P* < 0.001). The liver STE value had the highest diagnostic performance for hepatic cirrhosis compared to other biomarkers of serum fibrosis; the area under the ROC curve was 0.899 when the best cut-off value was 14.57 kPa. The sensitivity, specificity, positive predictive value, negative predictive value, and diagnostic accuracy were 78.6, 84.3, 79.7, 83.3, and 81.8%, respectively.

**Conclusion:**

The liver STE value has a high correlation with the stage of hepatic fibrosis in children with BA. STE has a good diagnostic performance in evaluating cirrhosis before KPE in children with BA.

## Introduction

Biliary atresia (BA) is a progressive fibrotic obliterative bile duct disease of the intrahepatic and extrahepatic biliary system (1). The underlying factors causing the disease have not been fully elucidated. The incidence of BA varies among regions. Indeed, previous studies reported that the incidence of BA is 1:15,000 to 1: 20,000 in live births in North America and Western Europe, 1 in 10,000 in Japan, 1 in 5,000 in Taiwan, and French Polynesia is that with the highest (1:3,000) (2, 3). BA has three main clinical characteristics: jaundice lasting for more than 14 days, stools with a light color or clay stools and urine with a dark color. If left untreated, it inevitably leads to liver cirrhosis, and most patients die within the first 2 years of life (4).

Currently, Kasai portoenterostomy (KPE) is the first-line treatment for BA (5). If the child does not have an evident cirrhosis before the surgery, the extrahepatic biliary tract can be constructed by KPE to relieve the obstruction of the biliary tract to a certain extent, to drain bile, eliminate the jaundice, improve liver function, and prolong the survival time of the native liver. However, KPE should not be the first choice in children with evident cirrhosis, rather liver transplantation should be chosen (6–9). A study showed that the worse the stages of hepatic fibrosis in children during KPE, the worse the long-term effect (10). Therefore, it is of the utmost importance to accurately assess the stages of hepatic fibrosis before performing KPE.

Liver biopsy is still currently considered the gold standard for the preoperative diagnosis of hepatic fibrosis. However, it has several shortcomings, including being an invasive examination, the possibility of making regional sampling errors, the need of sedating the patients, and the bleeding complications that may occur after biopsy (11). Therefore, a non-invasive diagnostic method is urgently needed to assess hepatic fibrosis to avoid the risks associated with liver biopsy.

Hepatic fibrosis can be assessed by serum biomarkers and imaging techniques, both representing non-invasive methods. The most common serum biomarkers are aspartate aminotransferase and platelet ratio index (APRI). However, some results in the use of APRI to assess the severity of hepatic fibrosis in patients with BA are inconsistent (12). The preferred imaging methods for evaluating hepatic fibrosis in children include ultrasound elastography and MRI elastography. However, MRI elastography is expensive, requires a long examination time, and infants and young children need sedation (13). At present, a small number of studies show the feasibility of ultrasound elastography in the assessment of the stages of hepatic fibrosis in pediatric patients with BA (14–17), but the results are different with different ultrasound elastography techniques, and the number of patients included in each study is small.

Mindray sound touch elastography (STE) technology is a new and promising non-invasive assessment method for hepatic fibrosis. It uses a queue-type emission focusing technology based on acoustic radiation force pulses, combined with the ultra-high frame rate and ultra-wide beam tracking detection technology of the domain platform. It is also supplemented by quality control tools such as respiratory stability and credibility graphs that can quickly and accurately capture and measure the shear wave propagation, so as to obtain sensitive, stable and accurate elastic modulus values (18). As far as we know, no research is available involving STE to assess the stages of hepatic fibrosis in patients with BA before KPE. Therefore, in this work, STE technology was used to detect the hepatic stiffness in children with BA using the pathological diagnosis of BA as the gold standard, with the aim to explore the diagnostic performance of STE in the diagnosis of cirrhosis in children with BA before KPE. In addition, STE performance was compared with that of transient elastography (TE) and serological markers.

## Materials and methods

### Participants

A total of 189 infants from the Hunan Children’s Hospital with highly suspected BA were selected in this study, and their preoperative liver STE values and related clinical data were prospectively collected from June 2018 to May 2021. The inclusion criterion was the following: patients were diagnosed with BA by intraoperative cholangiography and pathology. The exclusion criteria were the following: patients without BA excluded by cholangiography; patients whose guardians decided to abandon the treatment; patients whose guardians refused KPE and chose to wait for liver transplantation; patients whose guardians decide to choose a surgical treatment outside the hospital; patients with incomplete clinical data.

### Ethics statement

This study was approved by the ethics committee of Hunan children’s Hospital (approval No. HCHLL-2020-18) and was performed in accordance with the Declaration of Helsinki of 1975. Written informed consent was obtained from the guardians of each infant before the examination.

### Data collection

The following clinical data of the patients were collected: gender, age at KPE, body mass at KPE (BM), platelet count, aspartate aminotransferase (AST), gamma-glutamyl transpeptidase (GGT), and chitinase 3-like protein 1 (CHI3L1). The two clinical parameters of hepatic fibrosis APRI as well as gamma glutamyl transpeptidase-to-platelet ratio (GPR), were calculated according to GGT, AST, and PLT using the following formulas: APRI = AST (IU/L)/AST upper normal limit (IU/L) (=40)/platelet count (10^9^/L) × 100; GPR = GGT (IU/L)/GGT upper normal limit (IU/L) (=32)/platelet count (10^9^/L) × 100. All serological indicators were tested within 1 week before KPE.

### Ultrasound, sound touch elastography, and transient elastography examination

The sonography and STE examinations were performed on a Mindray Resona 7s scanner (Mindray Medical Systems, Shenzhen, China) by a radiologist with more than 10 years of experience in pediatric abdominal sonography and 5 years of experience in elastography. All scans were performed using a convex array transducer SC5-1U and a linear array transducer L14-5WU.

All infants were subjected to routine abdominal ultrasound examination within 3 days before KPE and fasted for at least 4 h. STE technology was used to assess liver stiffness measurement (LSM). Once the patient was asleep, the supine position was chosen to expose the upper abdomen. The scanning depth was set to 8 cm. One end of the probe rested on the costal arch, so that the costal arch supported most of the pressure of the probe, and the other end rested on the patient’s belly with the little finger of the hand holding the probe, in order to avoid pressure on the liver by the probe. The ultrasound beam was placed perpendicular to the part to be examined, the probe was slightly rotated or moved to avoid the gallbladder and make blood vessels visible. The STE program was run when the patient started to breath gently. The size of the sampling frame was adjusted to approximately 15 mm × 15 mm, and the sampling frame was placed 1 cm below the liver capsule of the right anterior lobe. The elastic scale was set to 0–60 KPa. The credibility graph and the motion stability button were turned on, and the image quality control was performed, which required more than 5 consecutive collections of elastic maps that meet the requirements on the same part. The image quality control requirements were the following (18): the color fullness in the sampling frame should be basically uniform, the reliability (RLB) index should reach 100%, the motion stability (M-STB) index ≥4 stars, and the SD ≤ 20% of the average. The diameter of the circular region of interest was 8–10 mm, measured five times, and the average was used for statistical calculations. The above operations were performed by the same radiologist who received ultrasound elastography training and had more than 5 years of experience in pediatric liver ultrasound elastography.

Transient elastography (Echosens, France) was used to assess hepatic stiffness and it was performed by an experienced operator. A probe (size S) was placed vertically on the skin surface of the right anterior lobe avoiding the main vessels. Ten values were obtained. A mean value was calculated as the final value, and the ratio of the interquartile range/median was <0.3.

### Evaluation of liver histology

Altogether, 159 surgical wedge biopsies were collected during KPE. The biopsies were fixed in formalin and embedded in paraffin. Hematoxylin and eosin staining was used to assess the morphology of the liver, while Masson’s trichrome staining or Van Gieson staining was used to assess the fibrosis stage of the liver. Two pathologists with more than 10 years of pediatric liver experience and blinded to the clinical patient data, observed and evaluated the stained slides until consensus was reached, otherwise another pathologist was involved in case of disagreement. The Metavir scoring system was used to evaluate the level of fibrosis. It consists of five stages, based on the architectural features of the portal fibrosis: F0 = no fibrosis, F1 = portal fibrosis without septa, F2 = portal fibrosis and few septa, F3 = numerous septa without cirrhosis, and F4 = cirrhosis.

### Statistical analysis

Statistical analysis was performed using the SPSS software (version 22.0, IBM Corp., Chicago, IL, USA) and MedCalc Statistical Software version 15.2.2 (MedCalc Software bvba^1^; 2015). Continuous variables were expressed as mean ± SD. The difference of the variables among groups at different fibrosis stages was analyzed by one-way analysis of variance (ANOVA) test and least-significant difference (LSD) *t*-test. The correlation of several variables with the fibrosis stage was analyzed using the Spearman correlation test. The logistic regression analysis was performed to understand the effects of ANOVA-significant variables on cirrhosis (F4). In addition, the diagnostic performance of those significant variables was assessed using the receiver operator characteristic (ROC) curve. The sensitivity, specificity, positive predictive value, negative predictive value, and area under the receiver operating characteristic curve (AUROC) were also calculated by the optimization of the Youden index. The optimal cutoff point was defined as the highest value of the Youden index. A *P*-value < 0.05 was considered statistically significant.

## Results

### Subject characteristics

**FIGURE 1:**
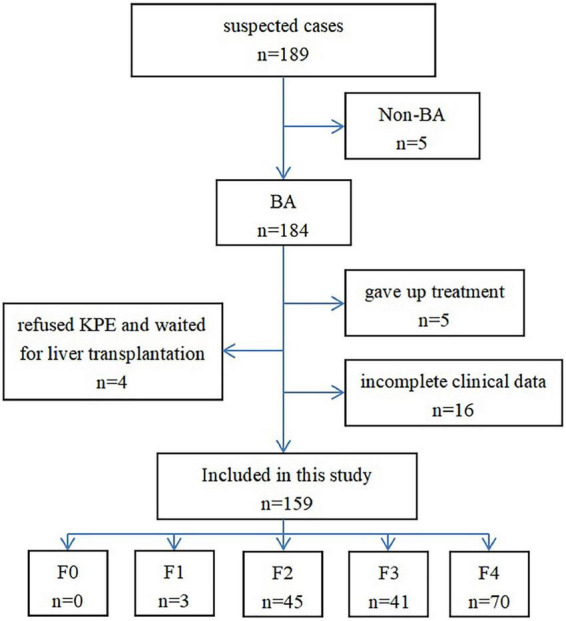
Flow chart of case selection in this study.

**FIGURE 2:**
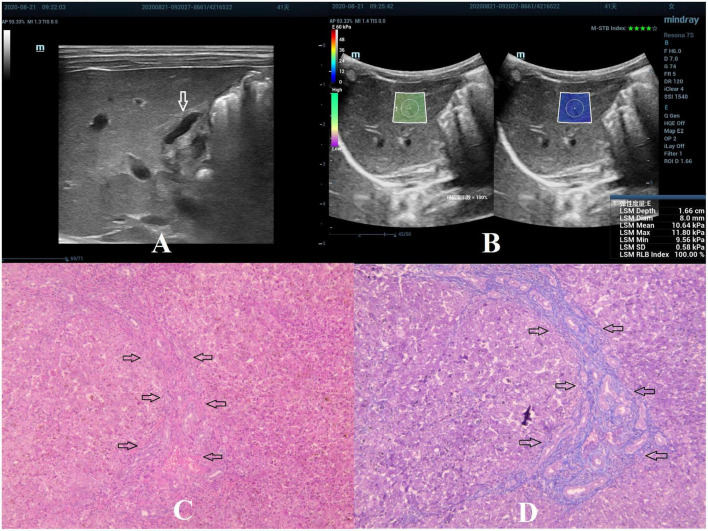
Biliary atresia in a 41-day-old girl with stage F2 hepatic fibrosis. **(A)** Arrow indicating the gallbladder abnormality. **(B)** STE image showing the mean value of liver stiffness of 10.64 Kpa. **(C)** Hematoxylin and eosin staining (40× magnification). The structure of the hepatic lobule was visible, the portal area was enlarged, and the bile canaliculi, blood vessels, and fibrous tissue proliferated (as indicated by the arrow). **(D)** Masson’s trichrome staining (40× magnification). The portal area was enlarged, and few septa were formed (as indicated by the arrow).

**FIGURE 3:**
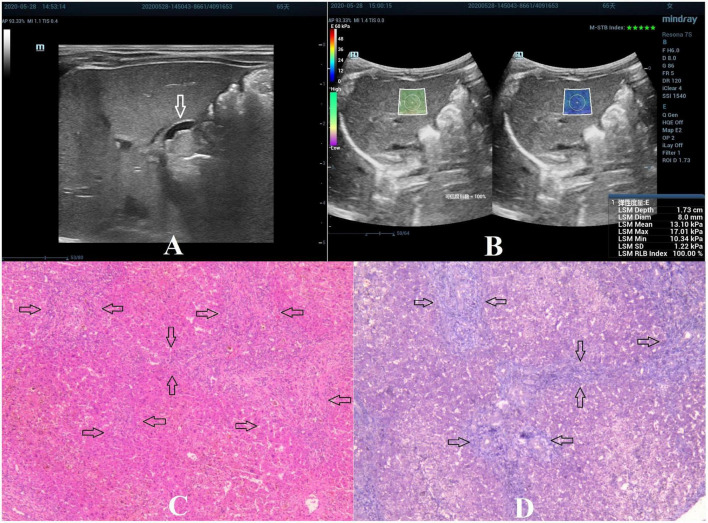
Biliary atresia in a 65-day-old girl with stage F3 hepatic fibrosis. **(A)** Arrow indicating the gallbladder abnormality. **(B)** STE image showing the mean value of liver stiffness of 13.10 Kpa. **(C)** Hematoxylin and eosin staining (40× magnification). The structure of the hepatic lobule was disordered, the bile canaliculi, blood vessels, and fibrous tissue proliferated in the portal area (as indicated by the arrow). **(D)** Masson’s trichrome staining (40× magnification). Significant proliferation of the fibrous tissue was found, forming numerous septa, but without pseudolobule formation (as indicated by the arrow).

**FIGURE 4:**
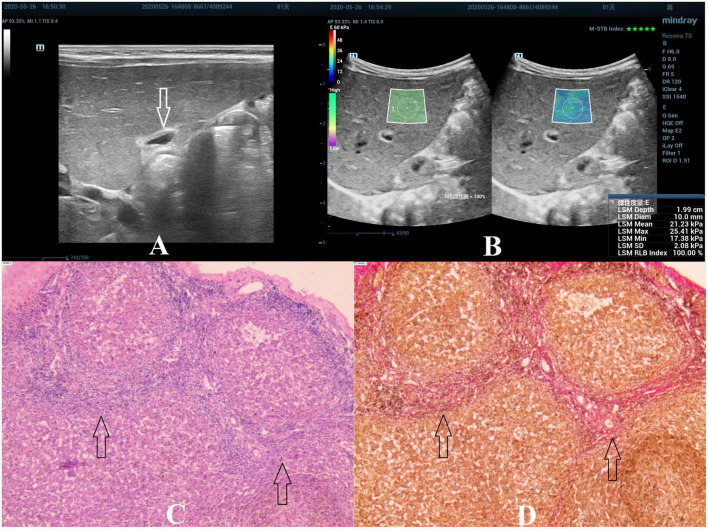
Biliary atresia in an 81-day-old boy with stage F4 hepatic fibrosis. **(A)** Arrow indicating the gallbladder abnormality. **(B)** STE image showing the mean value of liver stiffness of 21.23 Kpa. **(C)** Hematoxylin and eosin staining (40× magnification). The structure of the hepatic lobule disappeared, the fibrous tissue proliferated in the portal area, and the fibrous septa were formed, which surrounded the hepatocytes to form a pseudolobule. No normal central vein in the pseudolobule was found, and the structure of the hepatic sinusoids was disordered (as indicated by the arrow). **(D)** Van Gieson staining (40× magnification). Collagen fibers are stained red, and the pseudolobules are more clearly displayed (as indicated by the arrow).

Thirty cases were excluded from the 189 cases originally selected. Among them, 5 were diagnosed as non-BA by cholangiography, 5 abandoned the treatment, 4 refused KPE and waited for liver transplantation, and 16 did not have complete clinical data (Figure 1). Finally, 159 cases were included in this study, which included 65 males and 94 females; the age at the time of surgery was 35–124 days, and the median age was 56 days. The diagnosis of all the 159 patients with BA at different stages of hepatic fibrosis was made by pathologists, and included 3 patients at stage F1, 45 at stage F2, 41 at stage F3, 70 at stage F4, and none at stage F0 (Figures 2–4).

### Comparison of various parameters among different stages of hepatic fibrosis

**FIGURE 5:**
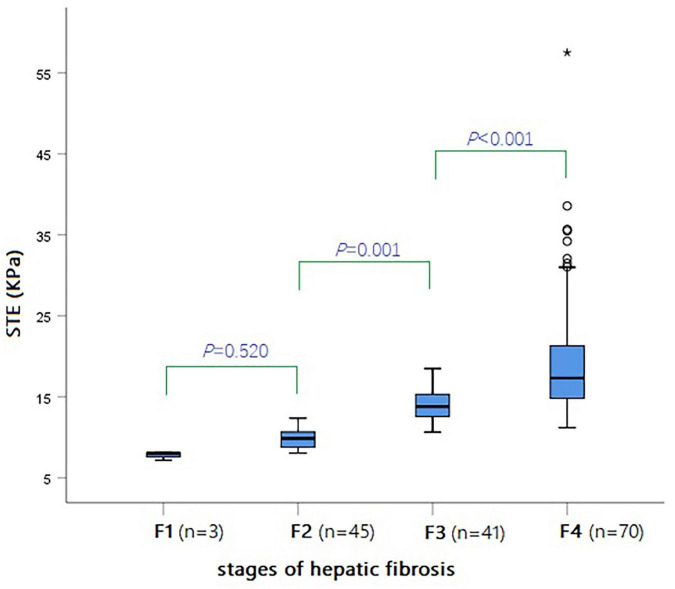
Box-and-whisker plot showing the STE values (KPa) for various stages of hepatic fibrosis in all BA patients.

The results of ANOVA analysis showed a statistically significant difference in the indicators of different hepatic fibrosis stages (all *P* < 0.05), except for CHI3L1 that was not statistically significant. LSD-*t* test was used for pairwise comparison, and the results showed that the STE value from F1 to F4 presented a gradually increasing trend. The continuous comparison of the STE values among the various fibrosis stages from F1 to F4 revealed a significant difference, except for F1 and F2 (Table 1 and Figure 5).

**TABLE 1:** Comparison of various parameters among different stages of hepatic fibrosis.

Variables	Stages	Mean ± SD (range)	ANOVA	LSD
				
			*P*-value	*P*-value*
Age at KP (d)	F1 (*n* = 3)	44.33 ± 4.62 (39–47)	<0.001	
	F2 (*n* = 45)	53.78 ± 10.90 (35–84)		0.291
	F3 (*n* = 41)	58.20 ± 13.14 (38–88)		0.173
	F4 (*n* = 70)	66.81 ± 18.04 (40–124)		0.004
AST (IU/L)	F1 (*n* = 3)	130.67 ± 20.67 (115.0–154.1)	<0.001	
	F2 (*n* = 45)	145.50 ± 54.16 (47.8–312.2)		0.75
	F3 (*n* = 41)	193.50 ± 82.57 (71.5–462.0)		0.005
	F4 (*n* = 70)	221.35 ± 88.14 (100.0–461.0)		0.071
PLT (10^9^/L)	F1 (*n* = 3)	540.67 ± 38.40 (518.0–585.0)	0.022	
	F2 (*n* = 45)	482.81 ± 161.57 (232.0–1,096.0)		0.499
	F3 (*n* = 41)	441.27 ± 126.32 (183.0–719.0)		0.181
	F4 (*n* = 70)	404.04 ± 142.03 (173.0–933.0)		0.188
GGT (IU/L)	F1 (*n* = 3)	137.60 ± 21.30 (113.0–150.0)	<0.001	
	F2 (*n* = 45)	326.44 ± 204.70 (80.0–816.0)		0.239
	F3 (*n* = 41)	431.56 ± 247.72 (72.0–1,282.0)		0.071
	F4 (*n* = 70)	588.84 ± 314.94 (166.0–1,842.0)		0.003
APRI	F1 (*n* = 3)	0.61 ± 0.12 (0.53–0.74)	<0.001	
	F2 (*n* = 45)	0.79 ± 0.30 (0.31–1.67)		0.655
	F3 (*n* = 41)	1.16 ± 0.51 (0.44–2.81)		0.015
	F4 (*n* = 70)	1.55 ± 0.93 (0.33–5.88)		0.005
GPR	F1 (*n* = 3)	0.75 ± 0.11 (0.68–0.90)	<0.001	
	F2 (*n* = 45)	2.36 ± 1.69 (0.53–7.97)		0.222
	F3 (*n* = 41)	3.20 ± 2.12 (0.73–12.39)		0.071
	F4 (*n* = 70)	4.79 ± 2.41 (1.32–11.32)		<0.001
CHI3L1 (ng/ml)	F1 (*n* = 3)	69.50 ± 35.67 (28.9–96.3)	0.503	
	F2 (*n* = 45)	52.83 ± 20.15 (17.6–102.4)		
	F3 (*n* = 41)	63.42 ± 40.58 (14.5–166.4)		
	F4 (*n* = 70)	59.12 ± 37.99 (11.7–172.0)		
STE (KPa)	F1 (*n* = 3)	7.77 ± 0.54 (7.15–8.13)	<0.001	
	F2 (*n* = 45)	9.91 ± 1.27 (8.05–12.36)		0.52
	F3 (*n* = 41)	14.01 ± 1.94 (10.64–18.48)		0.001
	F4 (*n* = 70)	19.99 ± 8.14 (11.19–57.50)		<0.001
TE (KPa)	F1 (*n* = 3)	10.53 ± 0.49 (10.20–11.10)	<0.001	
	F2 (*n* = 45)	11.16 ± 2.01 (6.60–15.00)		0.886
	F3 (*n* = 41)	13.82 ± 2.53 (9.57–20.80)		0.097
	F4 (*n* = 70)	19.91 ± 10.75 (8.10–45.00)		<0.001
BM (Kg)	F1 (*n* = 3)	4.26 ± 0.36 (3.85–4.47)	<0.001	
	F2 (*n* = 45)	4.37 ± 0.53 (3.01–6.00)		0.797
	F3 (*n* = 41)	4.59 ± 0.78 (3.10–6.00)		0.125
	F4 (*n* = 70)	4.65 ± 0.70 (3.50–7.00)		0.019

ANOVA, one-way analysis of variance test; LSD, least-significant difference *t*-test.

*Comparing STE between the previous group and the group considered.

### Correlation of various variables with hepatic fibrosis stages

**FIGURE 6:**
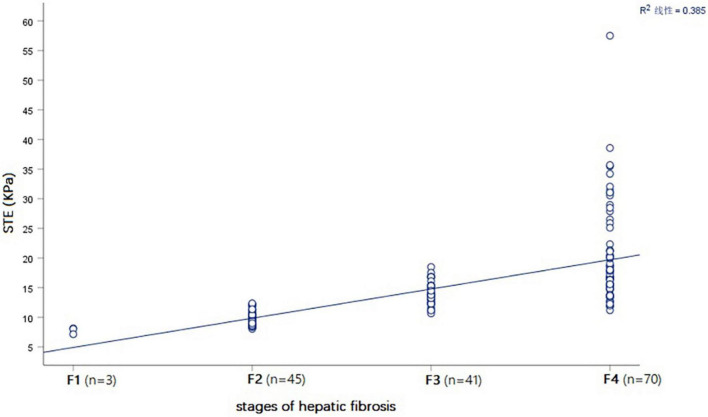
Spearman correlation plot showing that that STE value (KPa) was positively correlated with the stages of hepatic fibrosis in all BA patients.

Spearman correlation analysis showed that STE in children with BA had the highest correlation with the hepatic fibrosis stages, with a correlation coefficient *r* = 0.813 (*P* < 0.001) (Table 2 and Figure 6).

**TABLE 2:** Correlation of various variables with hepatic fibrosis stages.

Variables	Mean ± SD (range)	*r*	*P*-value
STE (KPa)	15.36 ± 7.06 (7.15–57.50)	0.813	<10.001
TE (KPa)	15.69 ± 8.27 (6.60–45.00)	0.555	<0.001
Age at KPE (d)	60.48 ± 16.00 (35–124)	0.354	<0.001
BM (Kg)	4.65 ± 0.70 (3.01–7.00)	0.342	<0.001
Sex (M/F)	65/94	0.139	0.081
GGT (IU/L)	465.51 ± 291.74 (72.00–1,842.00)	0.443	<0.001
APRI	1.22 ± 0.76 (0.31–5.88)	0.501	<0.001
GPR	3.61 ± 2.39 (0.53–12.39)	0.519	<0.001
CHI3L1 (ng/ml)	58.64 ± 34.56 (11.70–172.00)	−0.026	0.746
AST (IU/L)	190.99 ± 83.72 (47.80–462.00)	0.398	<0.001
PLT (10^9^/L)	438.51 ± 146.37 (173.00–1,096.00)	−0.27	0.001

### Logistic regression analysis

The Logistic regression analysis revealed that the indicators that entered the ROC curve analysis included STE, TE, APRI, GPR, BM, and age at KPE (all *P*-values < 0.05).

### Analysis of the receiver operator characteristic curve of various parameters to distinguish cirrhosis (F4) in children with biliary atresia

**FIGURE 7:**
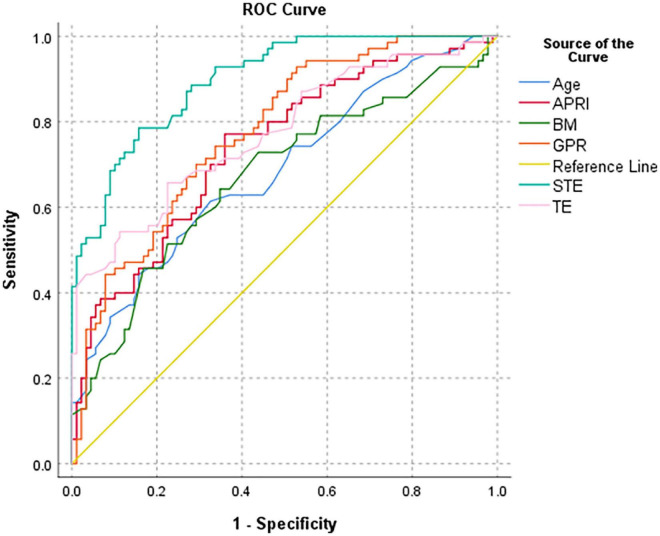
Receiver operator characteristic curve of various parameters to distinguish cirrhosis (F4) in all BA patients.

Among the various indicators, STE had the highest diagnostic efficiency for cirrhosis (F4) in infants with BA. The AUROC of STE was 0.899 and the optimal cut-off value was 14.57 kPa. The sensitivity, specificity, positive predictive value, negative predictive value, and diagnostic accuracy were 78.6, 84.3, 79.7, 83.3, and 81.8%, respectively (Table 3 and Figure 7).

**TABLE 3:** Diagnostic performance of various variables in children with biliary atresia with cirrhosis.

Variables	Area	*P*-value	Cut-off	*P*-value vs. STE	95% CI	Sensiti vity (%)	Specificity (%)	PPV (%)	NPV (%)	Accu racy (%)
STE (KPa)	0.899	<0.001	14.57	–	0.854, 0.944	78.60	84.30	79.70	83.30	81.80
TE (KPa)	0.773	<0.001	13.95	<0.001	0.699, 0.847	65.70	77.50	69.70	74.20	72.30
APRI	0.741	<0.001	0.96	<0.001	0.664, 0.818	77.10	64.00	62.80	78.10	69.80
GPR	0.777	<0.001	3.10	<0.001	0.706, 0.848	74.30	66.30	63.40	76.70	69.80
Age at KPE (d)	0.686	<0.001	57.50	<0.001	0.603, 0.769	61.40	67.40	59.70	69.00	64.80
BM (Kg)	0.672	<0.001	4.53	<0.001	0.587, 0.758	64.30	65.20	59.21	69.88	64.78

## Discussion

Our study revealed the role of STE with the Resona 7s Mindray Healthcare US system as a diagnostic tool to evaluate cirrhosis in BA. This study demonstrated that the STE values were in good correlation with the pathological stages of hepatic fibrosis in a large group of infants with BA. In addition, STE was superior to TE and serum fibrosis markers, such as APRI and GPR. Our study results showed the possibility to use STE technology to detect cirrhosis in infants with BA in a non-invasive manner, providing better management, feasible for clinical practice.

In recent years, several research reports have been published on the non-invasive assessment of hepatic fibrosis in children with ultrasound elastography. Chen et al. (14) used Aixplorer scanner (Supersonic Imagine) 2D Shear wave elastography (SWE) technology to measure jaundiced infants. The results showed that the 2D SWE value is positively correlated with the grade of hepatic fibrosis in children with BA, with a correlation coefficient of 0.789. The best cut-off value of SWE for diagnosing liver cirrhosis is 15.7 Kpa, the AUROC is 0.855, and the sensitivity and specificity are 75.0 and 77.8%, respectively. Galina et al. (7) used GE LOGIQ E9 US system SWE technology to detect liver stiffness in 19 infants with BA. The results showed that the best cut-off value for SWE to predict severe hepatic fibrosis and cirrhosis is 16 KPa, the AUROC is 0.98, and the sensitivity and specificity are 87.5 and 96.7%, respectively. Gao et al. (16) used Siemens Acuson S2000 acoustic radiation force impulse imaging technology to measure the shear wave velocity of the liver in 50 infants with BA. The results showed that it is positively correlated with the stage of hepatic fibrosis (*r* = 0.719). The best cut-off value for identifying liver cirrhosis is 2.16 m/s, the AUROC is 0.917, and the sensitivity and specificity are 87.5 and 90.5%, respectively. The results of our study showed that the higher the stage of hepatic fibrosis, the higher the liver stiffness value; the difference of STE values among different stages of hepatic fibrosis was statistically significant. Further Spearman correlation analysis showed that the STE value was positively correlated with the hepatic fibrosis stages, with a correlation coefficient of 0.813. This result suggested that STE had a high correlation with the liver stiffness of infants with BA and the stages of hepatic fibrosis; thus, STE could better reflect the stages of hepatic fibrosis in patients with BA. Our result also showed that the AUROC of STE to identify cirrhosis reached 0.899 in infants with BA. When the value of STE ≥14.57 kPa, the sensitivity, specificity, positive predictive value, negative predictive value, and diagnostic accuracy were 78.6, 84.3, 79.7, 83.3, and 81.8%, respectively, demonstrating the high diagnostic efficiency of STE for cirrhosis in infants with BA. Although the elastography technique used was different from that in the above article, the trends of the overall results were similar. The STE value has a high correlation with the stage of liver fibrosis and good diagnostic performance for cirrhosis, allowing us to perform STE examination in infants with BA to assess the stage of liver fibrosis before KPE without liver biopsy. This provided a quantitative reference for clinicians to assess the potential presence of liver cirrhosis before KPE. Combined with the age and other laboratory results of the children, STE could provide a quantitative reference for surgeons to choose the timing of surgery. On this basis, it was possible to continue performing STE examination after KPE, continuously monitoring the changes of liver fibrosis, and providing a quantitative reference for clinicians to predict the prognosis of patients with BA after KPE, as well as when liver transplantation is needed.

Previous studies used TE or Fibroscan as a non-invasive technique to assess hepatic fibrosis in children with BA. Shen et al. (12) used Fibroscan technology to study 31 patients with BA, and the results showed that LSM was positively correlated with the stage of hepatic fibrosis (*r* = 0.544), with an AUROC of LSM for F4 of 0.866. When the LSM ≥15.15 kPa, the sensitivity, specificity, positive predictive value, and negative predictive value were 85.7, 91.7, 75.0, and 95.7%, respectively. Our result showed that the TE technique had a relatively low diagnostic efficiency for BA cirrhosis as compared with the diagnostic efficiency showed in the results of Shen, which could be due to the difference in sample size. In addition, the results of our study suggested that TE is not as effective as the STE in the correlation of LSM with liver fibrosis staging, as well as the diagnostic performance of LSM for cirrhosis. The reason could be the different organizational incentive methods and data processing methods of the two approaches. TE is a probe that directly generates low-frequency shear waves and conducts them into the liver tissue, causing the deformation of the liver, which can be then detected, and the liver stiffness is displayed with a one-dimensional image. However, the probe in STE generates shear waves by emitting a focused ultrasound beam in the liver tissue and uses ultra-high frame rate and ultra-wide beam tracking technology to detect the propagation speed of the shear waves in the tissue to calculate the tissue hardness (19, 20), making this approach more precise.

Aspartate aminotransferase and platelet ratio index has been introduced as a useful non-invasive tool to evaluate hepatic fibrosis and BA prognosis. A study showed a significant positive correlation between APRI and the stage of fibrosis in infants with BA. The AUROC of APRI for diagnosing liver cirrhosis is 0.81. The sensitivity, specificity, and accuracy of APRI in diagnosing liver cirrhosis in children with BA when APRI ≥1.66, are 70.6, 82.7, and 82.4%, respectively (21). Our results are similar, although the diagnostic efficiency of our results was relatively low. The GPR, same as APRI, was calculated from two routine laboratory tests, which are widely available. A previous research shows that the GPR is useful in predicting the levels of hepatic fibrosis in chronic hepatitis B patients; the performance of GPR in predicting advanced fibrosis (≥F3) and cirrhosis (≥F4) is close to or higher than that of APRI (22). Our results showed that the correlation between GPR and hepatic fibrosis stage and its diagnostic performance for cirrhosis were similar to APRI. The reason could be due to the fact that the GPR model was too simple and was easily affected by various related factors. In addition, the age and body mass at KPE were positively correlated with the stage of fibrosis in infants with BA, but they had similarly lower diagnostic efficiency for cirrhosis.

Chitinase 3-like protein 1 is also called YKL-40 and its serum concentration is related to the stages of hepatic fibrosis (23). Huang et al. showed that serum CHI3L1 protein level can distinguish different stages of hepatic fibrosis in Chinese patients with hepatic fibrosis caused by HBV, being the best serological marker in the diagnosis of the stages of hepatic fibrosis (24). However, our result suggested that it is not highly correlated with the stages of hepatic fibrosis in children with BA. This result suggested that CHI3L1 might not be suitable for assessing the stages of hepatic fibrosis with BA.

This study has some limitations. Firstly, other factors, such as inflammation and edema, can also cause an increase in liver stiffness in addition to hepatic fibrosis. The inflammation in liver specimens was not considered when the relationship between STE and hepatic fibrosis was evaluated; thus, the STE value could not fully reflect the stages of hepatic fibrosis. Secondly, the possibility that STE values could replace age in the choice of surgical timing was not explored. More data will be collected in our future work, including patient outcomes, and the corresponding experimental methods will be designed to determine whether the STE values can replace the age when choosing the timing of surgery. Thirdly, STE could not be used to predict which cases would require liver transplantation because the considered set of data did not include those of liver transplant cases.

## Conclusion

In conclusion, liver stiffness detected by STE has a high correlation with the stage of hepatic fibrosis in children with BA. Thus, STE has a good diagnostic performance in the detection of cirrhosis and can be used to evaluate cirrhosis before KPE in children with BA.

## Data availability statement

The raw data supporting the conclusions of this article will be made available by the authors, without undue reservation.

## Ethics statement

The studies involving human participants were reviewed and approved by the Ethics Committee of Hunan children’s Hospital. Written informed consent to participate in this study was provided by the participants’ legal guardian/next of kin.

## Author contributions

XXD: data collection, design of the study, analysis and writing the manuscript, and project administration and funding acquisition. LY, RW, HC, HW, and WGL: data collection. LY and RW: oversee data collection. XXD and HXY: interpretation and manuscript review. All authors have read and agreed to the final version of the manuscript.

## Footnotes

1
http://www.medcalc.org
